# Acute injury characteristics predict chronic neuropathic pain development after spinal cord injury

**DOI:** 10.3389/fneur.2026.1814624

**Published:** 2026-06-16

**Authors:** Kenneth A. Fond, Mayra Arellano, Abel Torres-Espin, Austin Chou, Xuan Bradfield, Sara L. Moncivais, J. Russell Huie, Debra D. Hemmerle, Anastasia V. Keller, Vineeta Singh, Lisa U. Pascual, Anthony M. DiGiorgio, Jason F. Talbott, William D. Whetstone, Jonathan Z. Pan, Philip R. Weinstein, Sanjay S. Dhall, Rajiv Saigal, Adam R. Ferguson, Jacqueline C. Bresnahan, Michael S. Beattie, Nikos Kyritsis

**Affiliations:** 1 Weill Institute for Neurosciences, Brain and Spinal Injury Center, University of California, San Francisco (UCSF), San Francisco, CA, United States; 2 Department of Neurological Surgery, University of California, San Francisco (UCSF), San Francisco, CA, United States; 3 Zuckerberg San Francisco General Hospital and Trauma Center, San Francisco, CA, United States; 4 School of Public Health Sciences, University of Waterloo, Waterloo, ON, Canada; 5 Department of Neurology, University of California, San Francisco (UCSF), San Francisco, CA, United States; 6 Department of Orthopaedic Surgery, Orthopaedic Trauma Institute, University of California, San Francisco (UCSF), San Francisco, CA, United States; 7 Department of Radiology and Biomedical Imaging, University of California, San Francisco (UCSF), San Francisco, CA, United States; 8 Department of Emergency Medicine, University of California, San Francisco (UCSF), San Francisco, CA, United States; 9 Department of Anesthesia and Perioperative Care, University of California, San Francisco (UCSF), San Francisco, CA, United States; 10 Weill Institute for Neurosciences, Institute for Neurodegenerative Diseases, Spine Center, University of California, San Francisco (UCSF), San Francisco, CA, United States; 11 Harbor UCLA Medical Center, Torrance, CA, United States; 12 San Francisco Veterans Affairs Healthcare System, San Francisco, CA, United States

**Keywords:** biomarkers, neuropathic pain, predictive model, spinal cord injury, TRACK-SCI

## Abstract

**Introduction:**

Neuropathic pain is one of the most common and debilitating complications following spinal cord injury (SCI), frequently surpassing motor and sensory deficits as the symptom patients most want treated. Despite advances in understanding the molecular and physiological mechanisms underlying central neuropathic pain, effective treatments remain lacking and show wide variability in efficacy. Previous reports have indicated that early intervention represents the most effective pain management strategy, underscoring the clinical importance of identifying patients at risk during the acute care phase.

**Methods:**

We utilized the TRACK-SCI prospective clinical research database to assess neuropathic pain outcomes in all enrolled SCI patients and identify acute care variables predictive of chronic neuropathic pain development. Pain status was evaluated at 6 and 12 months post-injury. Candidate predictors were analyzed using multidimensional analytics, and a logistic regression model was constructed and validated using repeated 5-fold cross-validation.

**Results:**

Of 61 patients in the study cohort, 36 (59%) reported neuropathic pain in the chronic stages after SCI. Four acute care variables were identified as significant predictors of chronic neuropathic pain development: (1) the total number of systemic injuries sustained, (2) the injury severity score (ISS), (3) the lower limb total motor score, and (4) the sensory pinprick total score. The logistic regression model achieved a balanced accuracy of 74.3%, and repeated 5-fold cross-validation yielded an AUC of 0.708.

**Discussion:**

These findings highlight a crucial role of polytrauma in the development of chronic pain after SCI. The four identified predictors are parameters routinely measured in every trauma center, making the proposed model readily translatable to clinical practice. This predictive tool may enable earlier, targeted intervention for at-risk patients, addressing the clinical need for proactive pain management strategies in the acute post-SCI setting. Future work should validate this model in larger, independent cohorts and explore its utility in guiding early treatment decisions.

## Introduction

Spinal Cord Injury (SCI) is a catastrophic condition. Although it occurs in a relatively small percent of the United States (US) population, its impact can be profound. As of 2025, approximately 309,000 US residents live with SCI, with an estimated 18,421 new cases occurring annually (1). SCI involves a primary injury, often characterized by extensive neuronal cell death and immediate neurological consequences, as well as subsequent injuries caused by inflammation, excitotoxicity, and cellular damage, leading to sensory loss below the injury level (2, 3). These subsequent injuries typically manifest in later sub-acute or chronic phases. Despite their significant impact on quality of life, research on these secondary effects has been disproportionately limited (4, 5). Organizations such as the North American Spinal Cord Injury Consortium (NASCIC) have emphasized the need for SCI researchers to focus on reducing the burden of these complications (6).

Chronic pain after SCI is generally categorized into two main types: nociceptive pain, caused by inappropriate activation of peripheral pain receptors, and neuropathic pain, resulting from damage to central somatosensory nerve cells or fibers (7–10). Recently, the International Association for the Study of Pain (IASP) introduced a third category, nociplastic pain, which arises from altered pain perception without tissue damage and is distinct from neuropathic pain (11, 12). Neuropathic pain often persists into the chronic phase after SCI, severely impacting patients’ quality of life. It is commonly described using terms like “burning, cold, prickling, tingling, pins-and-needles, stabbing, shooting, lancinating, tight, swollen, and squeezing sensations.” Several studies estimate a median prevalence around 50% within 1 year of injury (12–15). A meta-analysis by Salehian et al. (15) reports a pooled prevalence of 57% (95% CI 51–64%) across 24 studies and 6,318 participants. In a 2012 study, 17% of patients with neuropathic pain rated their quality of life as “worse than death” (16). Other surveys indicate that managing chronic pain ranks among the highest priorities for SCI patients, along with regaining sexual function and restoring upper extremity function (17). Additionally, managing chronic pain is cited as the top daily challenge for these patients (18, 19). Due to the low prevalence of SCI, the pathophysiology of SCI-induced neuropathic pain remains poorly understood, making it difficult to diagnose and treat (20–24).

Treatment guidelines for neuropathic pain aim to (1) address underlying causes, (2) alleviate pain, and (3) prevent interference with quality of life. Treatment options include physical therapy, medications such as anti-seizure or antidepressant drugs, opioids, nerve blocks, peripheral nerve stimulation, brain stimulation, and ablative surgical procedures (25–27). Multimodal therapies combining two or more treatments are typically employed to manage neuropathic pain symptoms. However, these options vary in effectiveness and may carry significant side effects or complications. Research suggests that early diagnosis and intervention can alter the progression of neuropathic pain and provide valuable clinical insights (28, 29). We aim to utilize early diagnosis to administer preemptive treatment, which may reduce the severity of chronic neuropathic pain symptoms and mitigate the risk of progressing to later stages where advanced treatment with more significant side-effects is required. This underscores the importance of predicting neuropathic pain to facilitate early intervention.

Most clinical studies exploring “neuropathic pain,” “SCI,” “predictors,” and “biomarkers” take an approach driven by domain expertise, where researchers evaluate selected variables as potential biomarkers (14, 30, 31). Some studies also incorporate long-term follow-up with patients to identify predictors (32–34). Our clinical study takes a novel approach by leveraging a data-driven, unsupervised methodology. Using the comprehensive patient-level data from the Transforming Research and Clinical Knowledge in Spinal Cord Injury (TRACK-SCI) database, we developed a model to predict the onset of neuropathic pain. This process involved initial variable selection through unsupervised data analysis, supplemented by clinical expertise as needed. Predicting chronic neuropathic pain enables clinicians to implement preemptive treatments, educate patients, and refer them to specialized pain management services.

## Methods and materials

The Transforming Research and Clinical Knowledge in Spinal Cord Injury (TRACK-SCI) study is an Institutional Review Board (IRB)-approved, multi-center, prospective study of SCI (35). All procedures for this study were conducted with the approval of the Human Subjects Review Boards at the University of California at San Francisco (UCSF) and the U.S. Department of Defense (DoD) Human Research Protection Office. All activity relevant to the study was performed in accordance with relevant guidelines and regulations.

Eligibility criteria included all English- and non-English-speaking patients presenting to the emergency department (ED) with a traumatic SCI diagnosis. Exclusion criteria consisted of patients who were under 18 years of age, incarcerated, pregnant, on psychiatric hold, or in custody. Informed consent was obtained for all participants. For patients unable to provide consent due to their injury, an unaffiliated witness participated in the consent process, signing on behalf of the patient. When available, legally authorized representatives (LARs) or other suitable surrogates initially consented for these patients, who were subsequently approached for direct consent when possible. Participants could opt to take part in any combination of the following study components: blood draws, International Standards for Neurological Classification of Spinal Cord Injury (ISNCSCI) examinations, and follow-up assessments. Compensation of $50 was provided at each time point (hospital stay, 3-month phone call, 6-month in-person visit, 12-month in-person visit), up to a total of $200.

The foundation of the TRACK-SCI database is the National Institute of Neurological Disorders and Stroke (NINDS) recommended common data elements (CDEs) (36). Core CDEs are data elements that all SCI studies are strongly encouraged to use in collection of basic participant information. Additional measures from the International Spinal Cord Society (ISCoS) were also used. Data domains include demographic, clinical, radiologic, and functional outcome measures, stored in a Research Electronic Data Capture (REDCap) database (37). The database spans over 22,000 data points per patient, encompassing trauma characteristics, injury severity, blood pressure management, surgical interventions, hospital outcomes, high-frequency vital sign monitoring, motor-sensory exams, and pain assessments. Data were extracted from paper and electronic medical records, as well as participant interviews.

### Data curation

All data curation and analysis were done in the R programming language (38) using RStudio (39).

*Acute care data*: TRACK-SCI collects comprehensive data for each patient, totaling up to 22,000 data points from hospital admission to discharge (35, 40–43). These data are stored in a secure REDCap database. For our analysis, we excluded high-frequency vital monitoring variables (e.g., heart rate, mean arterial pressure) and details about medications used before the SCI or administered during the hospital stay. The only exception was pre-injury pain medication, which was retained in our dataset. Functional outcomes were evaluated using the ISNCSCI total subscores (motor and sensory) from the discharge examination. For patients lacking a discharge ISNCSCI exam, we used their most recent hospital-recorded examination.

The International Standards for Neurological Classification of Spinal Cord Injury (ISNCSCI) was utilized to assess motor and sensory functions and classify patients by injury severity using the American Spinal Injury Association (ASIA) Impairment Scale (AIS), ranging from A (complete, most severe) to E (normal, no impairment) (44). Variables like motor scores and sensory pinprick assessments were key components of the ISNCSCI exam. Trained clinical personnel, who had completed the ASIA International Standards Training E Program (InSTEP) and in-person training, conducted these examinations. ISNCSCI exams were performed during initial hospital admission, either as part of clinical care or separately for research purposes when ISNCSCI was not conducted clinically. In some cases, ISNCSCI exams could not be completed, often because patients were excessively sedated and could not participate in the exam. In such instances, assessors estimated the AIS grade based on available data and the patient’s clinical condition. If possible, patients were examined at regular intervals, including admission (day 0 = 0–23 h post-injury), every 24 h up to post-injury day 7, hospital discharge, 6-month follow-up (+/− 2 weeks), and 12-month follow-up (+/− 2 weeks). All ISNCSCI exam results were systematically recorded in the REDCap database.

*Neuropathic pain data*: Chronic pain data were derived from self-reported responses of TRACK-SCI-enrolled patients to two tools: the International Spinal Cord Injury Pain Basic Data Set (ISCIPBDS) Version 2.0 and a modified Douleur Neuropathique en 4 Questions (DN4) questionnaire (10, 45). These assessments were conducted via phone interviews at 3-, 6-, and 12-months post-SCI. Notably, DN4 outcomes from phone interviews have been shown to align closely with in-person assessments (46). To evaluate neuropathic pain, patients identified their three most severe pain problems, each of which was assessed individually with the DN4 questionnaire. A pain problem was classified as neuropathic if the DN4 score was ≥4. Patients were classified as having developed neuropathic pain if at least one of their reported pain areas was identified as neuropathic. Additionally, interviewers explained the characteristics of neuropathic and nociceptive pain, prompting patients to self-classify their pain into one of these categories. Generally, pain areas with DN4 scores ≥4 were also reported as neuropathic by the patients. In rare cases where DN4 scores and self-classifications conflicted, patient self-classification was given precedence.

### Data analysis

*Linear mixed-effects model*: A linear mixed-effects model was employed to analyze variations in neuropathic pain intensity scores across three post-SCI time points (3, 6, and 12 months). The model included both fixed effects (timepoint) and random effects to account for repeated measures within the same subjects. Specifically, a random intercept was used to allow each patient to have their own baseline pain intensity level, reflecting individual variability in pain perception and reporting. This approach accounts for differences in baseline pain intensity across patients while still modeling the overall population trends over time.

*Non-linear principal component analysis (NL-PCA)*: The final acute care dataset consisted of 63 variables from 61 patients. These patients were chosen based on the availability of pain data at 6- and/or 12-months post-SCI. To perform principal component analysis (PCA) and better understand how the variables correlated with one another, we first addressed the issue of missing data. Four of the 61 patients had missing data across the majority of the 63 variables and were excluded from further analysis. The remaining 57 patients had an overall missing data rate of 7.5% across all variables. To proceed with NL-PCA, we applied multiple imputation to estimate the missing values. Using the *mice* package (47) in R, we conducted 10 rounds of imputation with predictive mean matching (pmm), iterating 10 times per round. This process resulted in 10 complete datasets with no missing values. We then aggregated these 10 datasets by taking the median of the imputed values for numeric variables and the mode (most frequent imputed level) for categorical variables. NL-PCA was performed using the *Gifi* package (48) in R, employing the *princals* function with default parameters except for the number of computed dimensions, which was set to 10. All visualizations and permutation tests were generated using the *syndRomics* package (49) in R.

*Logistic regression*: We applied logistic regression using a combination of stepwise forward and backward model selection and domain expertise. This analysis focused on 20 variables identified from 1,000 permutation tests of the communalities of the first five principal components (PCs) with adjusted *p*-values < 0.1. First, we removed four categorical variables due to imbalanced observations across their levels. We then used the *stepAIC* function from the *MASS* package (50) to perform stepwise model selection based on the Akaike information criterion (AIC). Of the 16 remaining variables, only 10 had *p*-values < 0.05. This model achieved a balanced accuracy of 73.8% with a *p*-value of 0.009. Recognizing the potential challenges of implementing a 10-variable model in some clinical settings, we manually refined the model by sequentially removing variables and evaluating performance in terms of accuracy and *p*-values. This process led to a reduced model that retained strong predictive performance while improving clinical feasibility. One noteworthy adjustment involved replacing the upper extremity motor score with the lower extremity motor score to enhance model generalizability. While the upper extremity motor score was informative in our cohort—where cervical injuries predominated (65.6%)—it is less relevant for thoracic injuries, which generally spare the upper extremities. This replacement did not affect model performance, ensuring applicability across datasets with a different distribution of spinal cord injury (SCI) levels. The response variable for all models was whether patients reported neuropathic pain at 6- or 12-months post-SCI. Neuropathic pain was defined as a self-reported classification of at least one of their top three major pain problems as neuropathic. For patients with pain data at both time points, we used the 12-month data. Model performance, including statistics and the confusion matrix, was analyzed using the *caret* package (51). Marginal effects plots were created with the *sjPlot* package (52), and receiver operating characteristic (ROC) analysis was conducted using the *ROCR* package (53).

## Results

**Figure 1:**
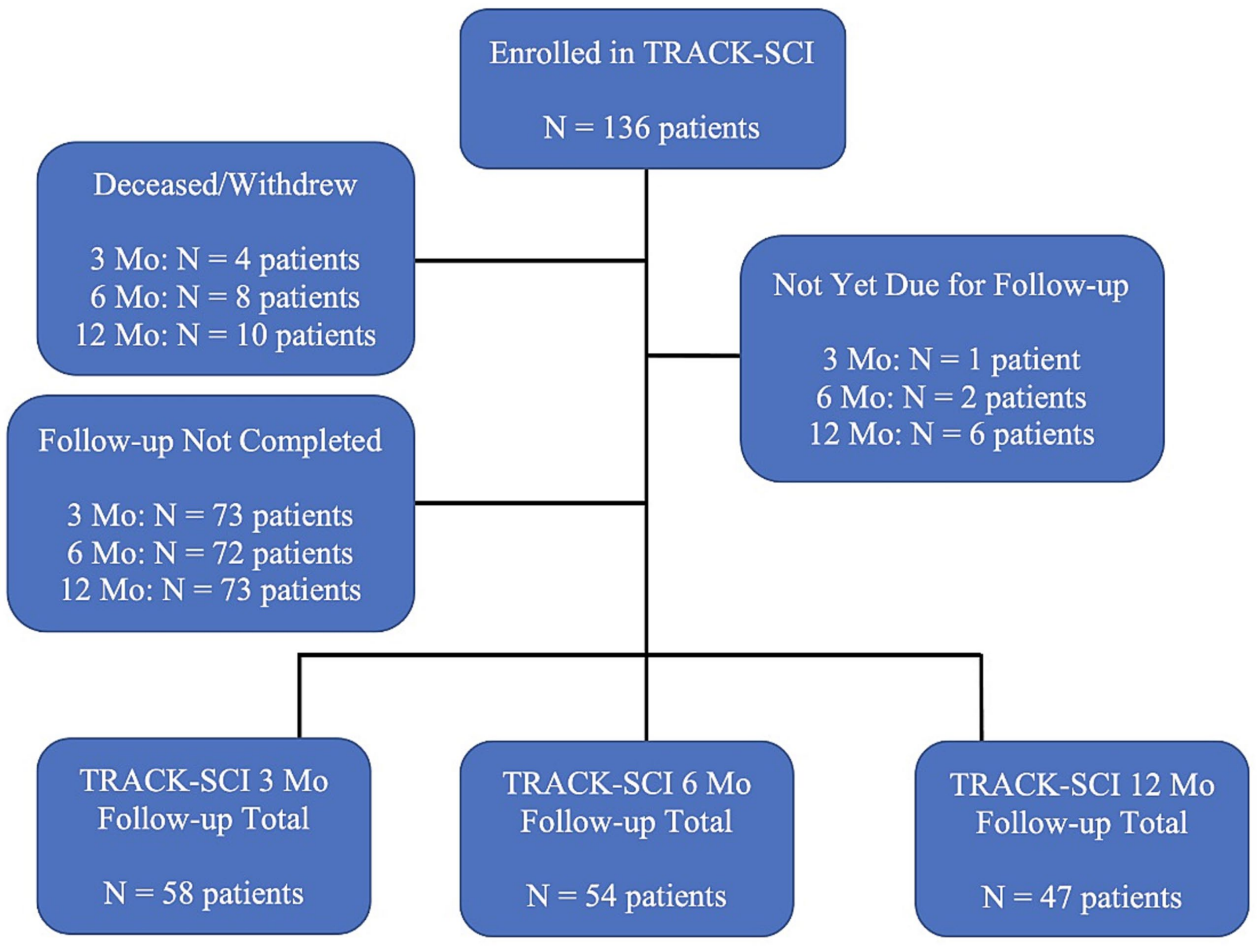
Logistic regression analysis suggests polytrauma and sensory pin-prick scores as important predictors of neuropathic pain development after SCI. **(A)** Out of 61 SCI patients with complete pain data, 36 reported they experience neuropathic pain at 6- and/or 12-months post SCI. The pain status (Yes/No) is the dependent variable in the logistic regression model. **(B–E)** Marginal effect plots of the four variables used in the logistic regression model. **(F)** Receiver operating characteristic (ROC) analysis of the model. The full model including all 61 patients has an area under the curve (AUC) of 0.793 (blue line). Repeated 1,000 times 5-fold cross validation (gray lines) has an average AUC of 0.708 (red line).

Our first objective was to analyze the TRACK-SCI database, which included 135 SCI patients enrolled at Zuckerberg San Francisco General Hospital and Trauma Center (ZSFG) from 2015 to 2021, with up to 22,000 data points per patient. Patient retention proved challenging during follow-up, particularly in the chronic phase of SCI. Some patients withdrew or were deceased, while many did not complete follow-up. At 3 months post-SCI, 58 patients completed follow-up, decreasing to 54 and 47 patients at 6 and 12 months, respectively (Figure 1). This decline reflects the difficulty of maintaining follow-up in a setting where acute rehabilitation occurs at separate facilities.

**Figure 2:**
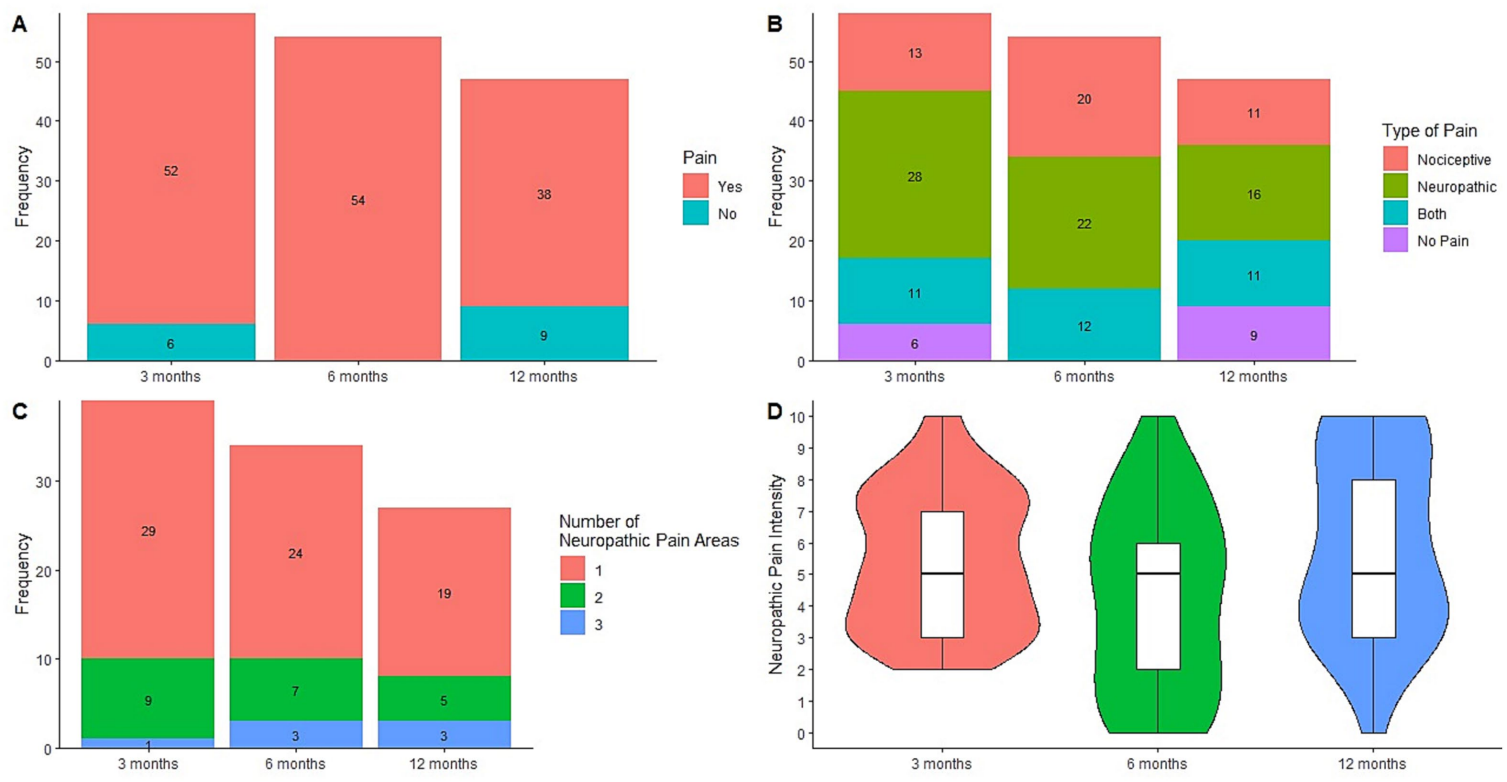
TRACK-SCI patient flow chart. By the time this analysis started, 136 SCI patients had enrolled in the TRACK-SCI study. Patients were followed into the chronic stages of SCI, at 3-, 6-, and 12-months post injury and their pain status was determined through self-reported questionnaires. As often happens in clinical studies the drop-out rate was significant with 58, 54, and 47 completed pain questionnaires at 3-, 6-, and 12-months post injury, respectively.

Pain status was assessed at each timepoint using pain questionnaire data. Over 80% of patients reported experiencing pain at all timepoints (89.7, 100, and 80.9% at 3, 6, and 12 months post-SCI, respectively; Figure 2A). Pain was categorized as nociceptive, neuropathic, or mixed. Neuropathic pain was the most frequently reported, present in 75, 63, and 71.1% of patients at 3, 6, and 12 months post-SCI, respectively (Figure 2B). This confirmed findings from previous studies.^21^ Most patients reported one neuropathic pain area (74.4, 70.6, and 70.4% at 3, 6, and 12 months, respectively), with a few reporting up to three areas of neuropathic pain (Figure 2C). Pain intensity scores, rated on a 0–10 scale, were assessed for each neuropathic pain area, and they varied across timepoints (5.46 ± 2.18, 4.19 ± 2.91, and 5.93 ± 2.94 for 3, 6, and 12 months, respectively). The 6-month time point showed slightly reduced pain intensity, which was statistically significant (linear mixed-effects model, *p* = 0.009). However, the fact that the overall distributions appeared similar, and the number of cases varied across the timepoints, do not allow more in-depth interpretations of these data (Figure 2D). Together, these data show pain manifestation in the TRACK-SCI enrolled patients over time suggesting only minor differences in type and intensity between the assessed timepoints.

One of the major goals of our study is to determine whether we can predict the development of neuropathic pain after SCI using data collected acutely during the initial hospitalization. Because neuropathic pain usually appears within the first 6 months after SCI, we decided to predict neuropathic pain using data from the 6- and 12-months post SCI questionnaires. To increase our sample size, we collapsed the 6- and 12-month data into one set and used the latest data point available. Collapsing these two time points resulted in a cohort of 61 patients with pain data. We extracted the acute care data we had for these patients from the TRACK-SCI database. The initial dataset included 2,047 variables. Through data-driven methods and domain expertise (see Materials and Methods) we narrowed our dataset number of variables to 63 (Supplementary Figure 1). Even though the problem of missing data was addressed significantly during our variable selection process, there was still data missing in our final dataset. While a small percentage of missing data could be allowed for some analyses, we required a complete dataset for others and thus needed to impute the missing data points using multiple imputation by chained equations.

**Figure 3:**
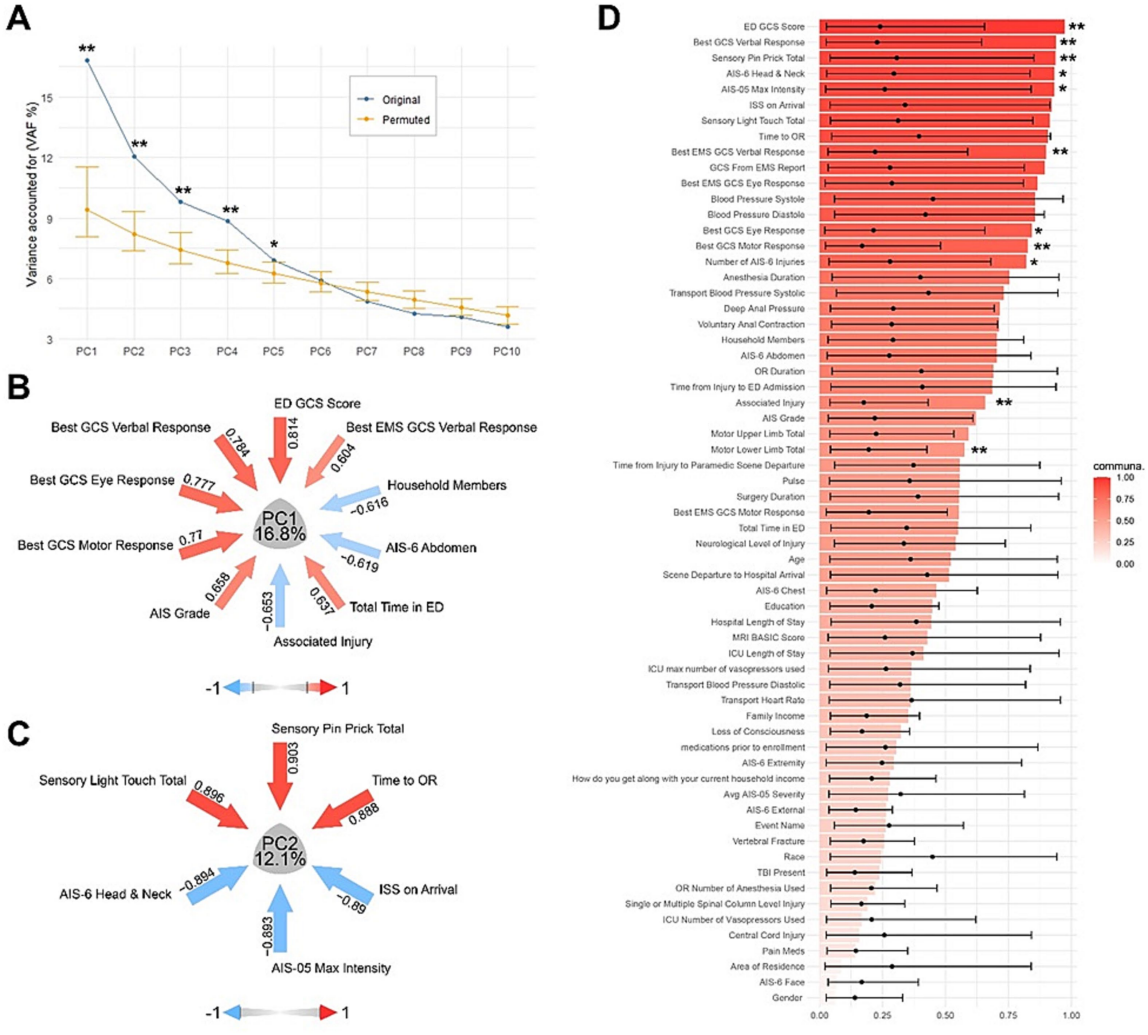
Chronic pain assessment of TRACK-SCI enrolled patients. **(A)** The vast majority of TRACK-SCI patients experience pain at 3-, 6-, 12-months post SCI. **(B)** From the SCI patients who reported pain in A, the most prevalent type of pain is neuropathic across all time points with a significant number of patients reporting both neuropathic and nociceptive pain. **(C)** Most patients experiencing neuropathic pain post SCI reported that the pain was confined in one area, with a few of them listing two areas and even fewer cases with three neuropathic pain areas. **(D)** The average pain intensity of the neuropathic pain areas did not change significantly over time.

We then used non-linear principal component analysis (NL-PCA), an unsupervised multivariate technique, to determine whether any combination of the variables (principal component; PC) or a combination of PCs could be used as predictors for chronic neuropathic pain development after SCI. First, we performed a NL-PCA and a permutation test (1,000 permutations) to determine how many PCs to use in downstream analyses. The permutation test results showed that the first 5 PCs were significantly different from random noise (Figure 3A), together accounting for 54.4% of the total variance. Using another permutation test for each individual variable, we determined which variables were significantly contributing (i.e., loading) to each one of these PCs. The first two PCs (28.9% of total variance), the ones with the most variables significantly contributing to, are visualized in Figures 3B,C. PC1 represents an overall clinical picture of the patients in the emergency department, while PC2 appears to reflect on pain severity and sensation. The standardized loadings of all 63 input variables on PC1–PC5, together with permutation-based statistical significance for each variable-PC pairing, are shown in Supplementary Figure 2. However, using these two PCs alone, together, or in various combinations with the remaining three PCs as predictors in a PC-only logistic regression model was not sufficient to accurately predict the development of neuropathic pain (data not shown). Since the PCs were not good predictors for neuropathic pain, we decided to use the PCA outcome as a method to perform unsupervised variable selection. We calculated the variance of each variable captured (communalities) in the first 5 PCs (Figure 3D) and assessed their significance above random chance by a permutation test (1,000 permutations). We then selected the most important variables (*p* < 0.1, *p* = 20) from the permutation of communalities to be used in a logistic regression model to predict the presence of neuropathic pain.

**Figure 4:**
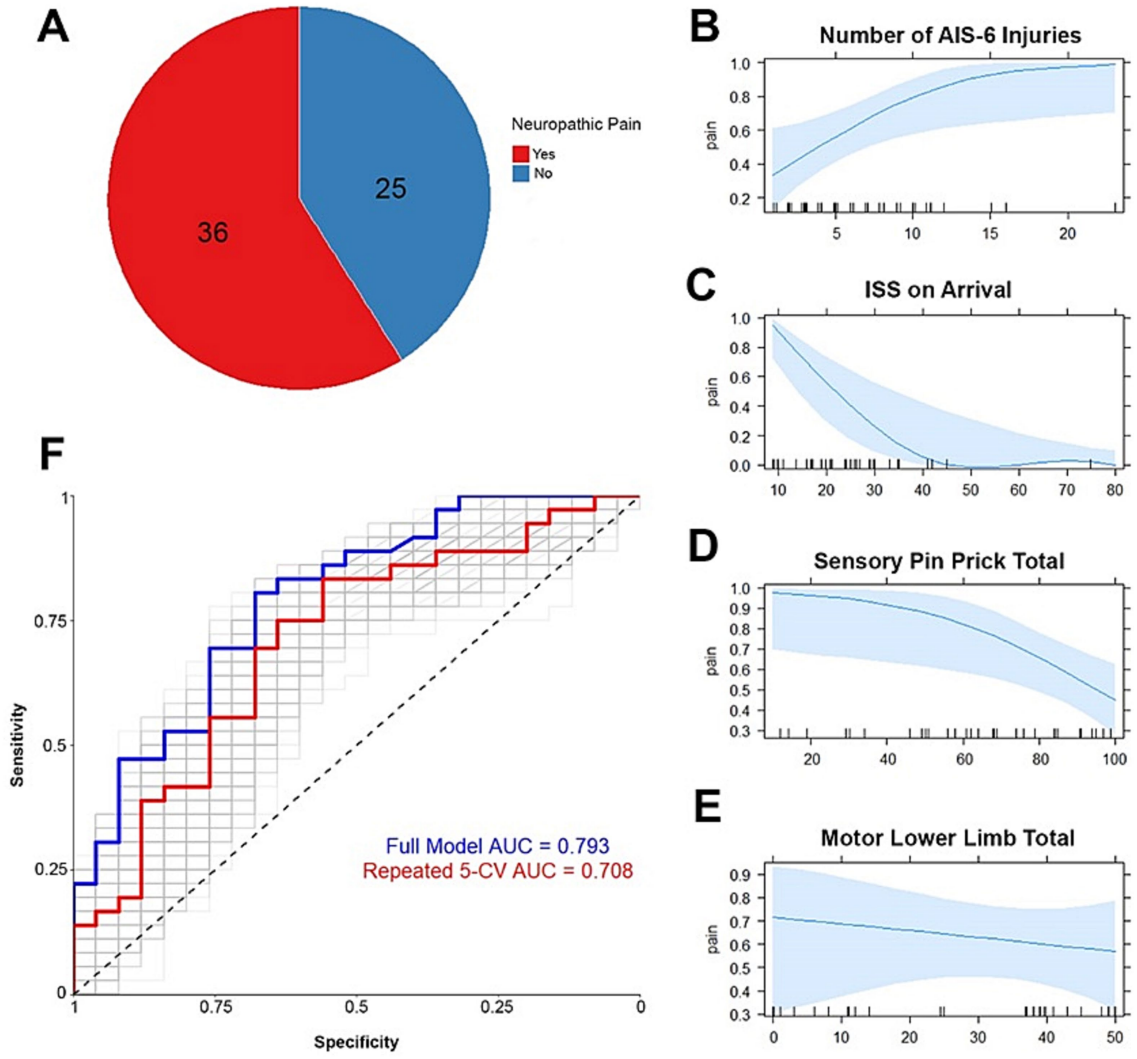
Principal component analysis and permutation analysis is used to rank the acute care variables. **(A)** Nonlinear PCA analysis of the 63 acute care variables followed by 1,000 permutation tests reveal 5 stable Principal Components (PCs) that account for 54.4% of the total variance. **(B,C)** The highest loading variables in PC1 appear to be related to the ED clinical picture of the patients, and the PC2 variables represent pain severity and sensation. **(D)** One thousand permutation tests on the communalities of the first 5 PCs were used to rank the importance of the variables and measure their stability. *p < 0.05, **p < 0.01 (GCS, Glasgow Coma Scale; AIS, ASIA Impairment Scale; ISS, Injury Severity Score).

As mentioned, we have pain data at chronic time points (6- and/or 12-months post SCI) from 61 patients, 36 of which reported neuropathic pain (Figure 4A and Table 1). Stepwise forward and backward selection was used in combination with domain expertise to determine a parsimonious logistic regression model (Figures 4B–E and Table 2). The overall accuracy of the model was 75.4% (balanced accuracy 74.3%) with a *p*-value of 0.006 (Supplementary Figure 3). Our model has not been tested in an independent and external dataset. To assess the validity of our model, and test the possibility of overfitting the data, we performed 5-fold cross-validation, which was repeated 1,000 times. Our full model had an AUC of 0.793, the repeated cross-validation gave an average AUC of 0.708 (Figure 4F).

**Table 1:** Demographic and additional important clinical information of the 61 patients with complete pain data at 6- and/or 12-months post SCI.

	Yes (*N* = 36)	No (*N* = 25)	Total (*N* = 61)
Gender			
Female	11 (30.6%)	4 (16.0%)	15 (24.6%)
Male	25 (69.4%)	21 (84.0%)	46 (75.4%)
Race			
American Indian or Alaska Native	1 (2.8%)	0 (0%)	1 (1.6%)
Asian	7 (19.4%)	7 (28.0%)	14 (23.0%)
Black or African-American	6 (16.7%)	2 (8.0%)	8 (13.1%)
Hispanic	4 (11.1%)	3 (12.0%)	7 (11.5%)
Native Hawaiian or Other Pacific Islander	1 (2.8%)	1 (4.0%)	2 (3.3%)
White	16 (44.4%)	10 (40.0%)	26 (42.6%)
Other	0 (0%)	2 (8.0%)	2 (3.3%)
Unknown	1 (2.8%)	0 (0%)	1 (1.6%)
Age			
Mean (SD)	55.3 (17.6)	54.8 (19.9)	55.1 (18.4)
Median [Min, Max]	54.5 [21.0, 89.0]	59.0 [19.0, 84.0]	55.0 [19.0, 89.0]
AIS grade at discharge			
A	5 (13.9%)	2 (8.0%)	7 (11.5%)
B	2 (5.6%)	1 (4.0%)	3 (4.9%)
C	4 (11.1%)	5 (20.0%)	9 (14.8%)
D	22 (61.1%)	14 (56.0%)	36 (59.0%)
E	1 (2.8%)	1 (4.0%)	2 (3.3%)
Unknown	2 (5.6%)	2 (8.0%)	4 (6.6%)
Neurological level of injury			
Cervical	27 (75.0%)	13 (52.0%)	40 (65.6%)
Thoracic	1 (2.8%)	1 (4.0%)	2 (3.3%)
Lumbar	2 (5.6%)	2 (8.0%)	4 (6.6%)
Unknown	6 (16.7%)	9 (36.0%)	15 (24.6%)
Injury Severity Score (ISS)			
Mean (SD)	20.8 (8.61)	23.3 (13.1)	21.8 (10.6)
Median [Min, Max]	17.0 [10.0, 45.0]	21.0 [9.00, 75.0]	19.0 [9.00, 75.0]
Pain medications prior to SCI			
Yes	5 (13.9%)	6 (24.0%)	11 (18.0%)
No	31 (86.1%)	19 (76.0%)	50 (82.0%)

The terms “Yes” and “No” indicate neuropathic pain presence.

**Table 2:** Coefficient estimates of the logistic regression model.

Predictors	Pain		
	Log-odds	CI	*p*
(Intercept)	7.23	2.91–12.67	**0.003**
Sensory pin prick total	−0.04	−0.08 to −0.01	**0.017**
Motor lower limb total	-0.01	−0.06 to 0.04	0.602
Number of AIS 6 injuries	0.23	0.05–0.45	**0.024**
ISS on arrival	−0.18	−0.34 to −0.07	**0.009**
Observations	61		
*R*^2^ Tjur	0.256		

*p* < 0.05 appear as bold.

To assess whether model performance was uniform across clinically meaningful patient strata, we evaluated the locked logistic regression model within pre-specified subgroups defined by neurological level (cervical vs. thoracolumbar), age (< vs. ≥ median), AIS grade at discharge (A-B vs. C-E), sex, and ISS (< vs. ≥ median); per-subgroup performance metrics with bootstrap confidence intervals are provided in Table 3. Discrimination was broadly stable, with subgroup AUCs ranging from 0.71 to 0.85, excluding two cells with *n* < 15 (Thoracolumbar, AIS A-B), where the model classified all patients correctly, yielding AUC point estimates of 1.00 that reflect the absence of misclassifications in small cells rather than meaningful discrimination. Likelihood-ratio tests comparing nested models with and without a discrimination-by-subgroup interaction term confirmed no significant differential performance by neurological level (*p* = 0.35), age (*p* = 0.63), or sex (*p* = 0.41); a borderline trend was observed for AIS grade (*p* = 0.066), reflecting perfect classification in the small AIS A-B subgroup and warranting confirmation in larger cohorts. These analyses indicate that within this single-center cohort, model performance does not appear to be driven by any one clinical stratum, although external validation in geographically and demographically distinct cohorts remains essential.

**Table 3:** Performance of the locked four-variable logistic regression model within pre-specified clinical subgroups of the TRACK-SCI cohort (*n* = 61).

Subgroup	*n*	Events	Accuracy	Sens.	Spec.	AUC (95% CI)
Overall	61	36	0.75	0.81	0.68	0.79 (0.67–0.90)
Neurological level						
Cervical	40	27	0.73	0.74	0.69	0.77 (0.58–0.92)
Thoracolumbar*	6	5	0.83	1.00	0.67	1.00 (0.56–1.00)
Age						
< median (55 y)	29	18	0.69	0.78	0.55	0.74 (0.53–0.90)
≥ median (55 y)	32	18	0.81	0.83	0.79	0.85 (0.68–0.96)
AIS grade at discharge						
A-B*	10	7	1.00	1.00	1.00	1.00 (1.00–1.00)
C-E	47	27	0.68	0.74	0.60	0.71 (0.54–0.85)
Sex						
Male	46	25	0.76	0.80	0.71	0.82 (0.67–0.92)
Female	15	11	0.73	0.82	0.50	0.77 (0.46–0.96)
Injury Severity Score (ISS)						
< median (19)	30	20	0.70	0.75	0.60	0.75 (0.55–0.91)
≥ median (19)	31	16	0.81	0.88	0.73	0.85 (0.56–0.96)

*Performance estimates in subgroups with *n* < 15 (Thoracolumbar, AIS A-B) are based on perfect classification of the small number of patients in those cells; AUC point estimates of 1.00 should be interpreted as “the model made no errors in this small subgroup” rather than as evidence of perfect discrimination, and the wide bootstrap CI for thoracolumbar reflects the underlying uncertainty.

## Discussion

Neuropathic pain is one of the most significant consequences of spinal cord injury (SCI). Numerous reports and anecdotal accounts from SCI patients underscore the prioritization of neuropathic pain treatment over motor improvements, particularly in the long term (>6 years) (19). Despite substantial progress in understanding the mechanisms of neuropathic pain, effective treatments remain elusive (20, 33). Most patients are left to adapt and cope with persistent pain (6). Early intervention has emerged as a crucial strategy for neuropathic pain management, with studies indicating that patients who address their pain early achieve a higher quality of life than those who delay treatment (29, 51–54). This study leveraged acute care hospital data from patients with SCI to develop a model predicting the onset of neuropathic pain at 6- and 12-months post-injury. Advanced prediction models can facilitate early treatment initiation, significantly improving the quality of life for SCI patients.

Prior to analyzing acute care data, we examined the TRACK-SCI database to evaluate chronic pain prevalence, comparing our cohort with previously reported findings. Among 61 patients in our study, 36 (59%) reported neuropathic pain at 6 and 12 months post-SCI. This prevalence aligns with rates reported in similar studies: 40% (55), 57.4% (32), 59% (56), and 69.1% (13). With the exception of Kim et al. (13), these figures fall within the 38.58–67.47% range proposed by Burke et al. (14), suggesting our cohort reflects known patterns of neuropathic pain prevalence. While previous studies consistently report similar neuropathic pain prevalence, no consensus was reached for which factors best predicted neuropathic pain. Variables such as marital status, pain adaptation, cold-evoked dysesthesia, older age, trauma cause, injury type, and early pain onset have been suggested as potential biomarkers. Our analysis, however, highlights acute polytrauma characteristics and motor and sensory scores from the ISNCSCI examination as key predictors of neuropathic pain development.

The number of total injuries, injury severity score, motor score, and sensory pinprick score are key measures of SCI severity and are associated with the risk of developing neuropathic pain. Greater injury severity likely contributes to increased nerve damage, exacerbating inflammation and disrupting neural pathways. These disruptions can trigger excitotoxicity and maladaptive plasticity, leading to heightened pain sensitivity (57–59). Reductions in motor and sensory pinprick scores reflect damage to motor and sensory pathways, which may result in abnormal neural signaling and altered receptor function, further contributing to neuropathic pain (23). Moreover, sensory profiles have been shown to influence central neuropathic pain development, with an imbalance between the spinothalamic (pain and temperature perception) and dorsal column (touch and vibration) pathways playing a significant role (60). This imbalance, along with structural damage and inflammation, underscores the complex mechanisms by which SCI leads to neuropathic pain development.

In this study, pain intensity was assessed using a self-reported numeric rating scale (NRS) from 0 to 10, where patients rated the severity of their neuropathic pain for each reported pain area. While the NRS is technically an ordinal scale that could justify a different type of analysis (61), it is commonly treated as an interval scale in pain research to facilitate statistical analysis. This treatment is based on the assumption that the intervals between points on the NRS are perceived as equidistant by patients, allowing for parametric tests that offer greater statistical power. However, it is important to acknowledge the limitations of this approach. For completeness, the use of the NRS as an interval scale in this study is justified by the need to assess longitudinal trends in pain intensity across time points. Although we found a statistically significant reduction in pain intensity at the 6-month time point, with a mean change of approximately 1.3 points (*p* = 0.009), this change does not meet the generally accepted threshold of 2 points for clinical relevance (62, 63). Future studies should continue to evaluate both statistical and clinical significance when interpreting changes in pain intensity.

The assessment of neuropathic pain areas also required careful methodological consideration. Patients were asked to identify their three worst pain problems, each of which was evaluated using both the International Spinal Cord Injury Pain Basic Data Set (ISCIPBDS) and the DN4 questionnaire. By employing standardized tools and guided interviews, we ensured consistency in identifying and characterizing neuropathic pain areas across time points. Nonetheless, potential variability in patient reporting and recall must be acknowledged. The longitudinal design of this study mitigated some of these limitations by focusing on consistent methods of pain evaluation.

One methodological decision in this study was the collapsing of 6- and 12-month data to increase the sample size for the logistic regression model. This approach is supported by the chronic nature of neuropathic pain, which is unlikely to resolve spontaneously once established in the chronic phase. Retaining the latest available data point for each patient ensures that the analysis reflects clinically relevant outcomes while enhancing statistical power. While this decision introduces limitations in predicting the precise timing of pain onset, it remains a practical solution given the constraints of sample size and follow-up data availability.

Additionally, the model was developed and cross-validated using data from a single hospital (ZSFG). Although our parsimonious model selection and repeated cross-validation (1,000 iterations) minimize overfitting concerns and demonstrate robust performance despite some decline in AUC, independent validation is essential for confirming generalizability. Additional limitations include a high dropout rate, small sample size, and missing data—challenges commonly faced in SCI studies. Furthermore, reliance on self-reported pain data introduces subjectivity.

A major strength of our model lies in its reliance on variables routinely collected in every trauma center. As TRACK-SCI continues to enroll patients, additional cohorts can be used for further validation. Simultaneously, colleagues at other centers can assess and refine our model. Expanding and validating this approach holds immense potential for improving patient outcomes by enabling clinicians to predict chronic neuropathic pain during the acute phase post-injury. This predictive capability opens a critical window for preemptive interventions, ultimately enhancing quality of life for individuals with SCI. Our findings suggest that commonly collected variables at level I trauma centers can effectively predict chronic neuropathic pain, paving the way for early treatment and improved long-term outcomes for SCI patients.

## Data Availability

The datasets presented in this study can be found in online repositories. The names of the repository/repositories and accession number(s) can be found at: https://doi.org/10.34945/F5VC85.
