# Supplementary material for: Acute injury characteristics predict chronic neuropathic pain development after spinal cord injury
Source: Front Neurol. 2026 Jun 16;17:1814624. doi: 10.3389/fneur.2026.1814624 (PMC13314459; doi:10.3389/fneur.2026.1814624)
Supplement: Supplementary file 1 [file Table_1.DOCX]

**SUPPLEMENTARY FIGURES**


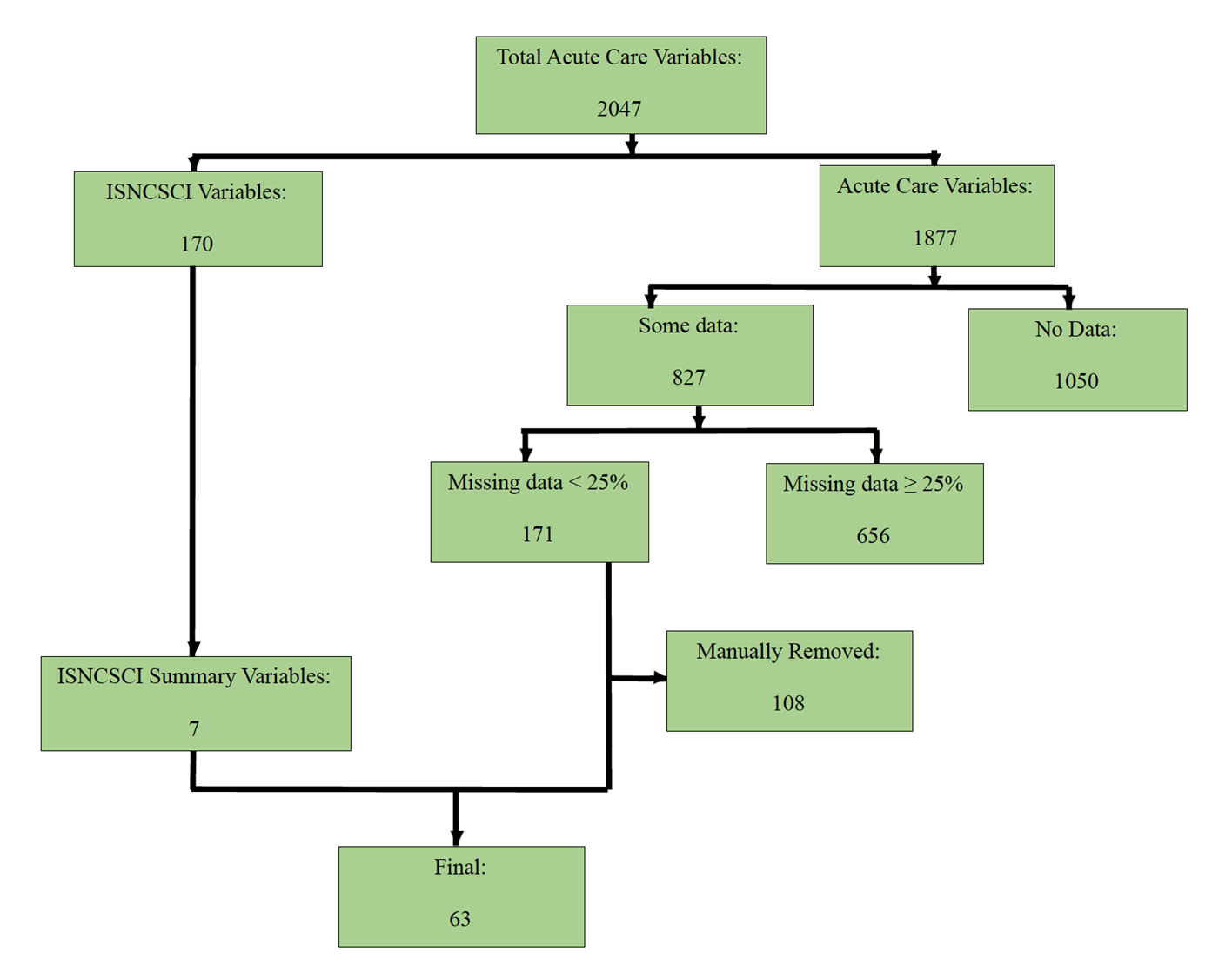


**Supplementary Figure 1. Flow chart of the acute care variable selection as potential predictors for the development of chronic neuropathic pain.** Using the TRACK-SCI acute care database we started with 2,047 variables. Data curation process consisted of filtering out variables with over 25% missing data, manually removing variables the purpose of which was only for database maintenance, and selecting only the total scores of the INSCSCI examination at discharge (or latest at hospital). The final number of variables used for multivariate analysis was 63. (Abbreviations: AIS, ASIA Impairment Scale)


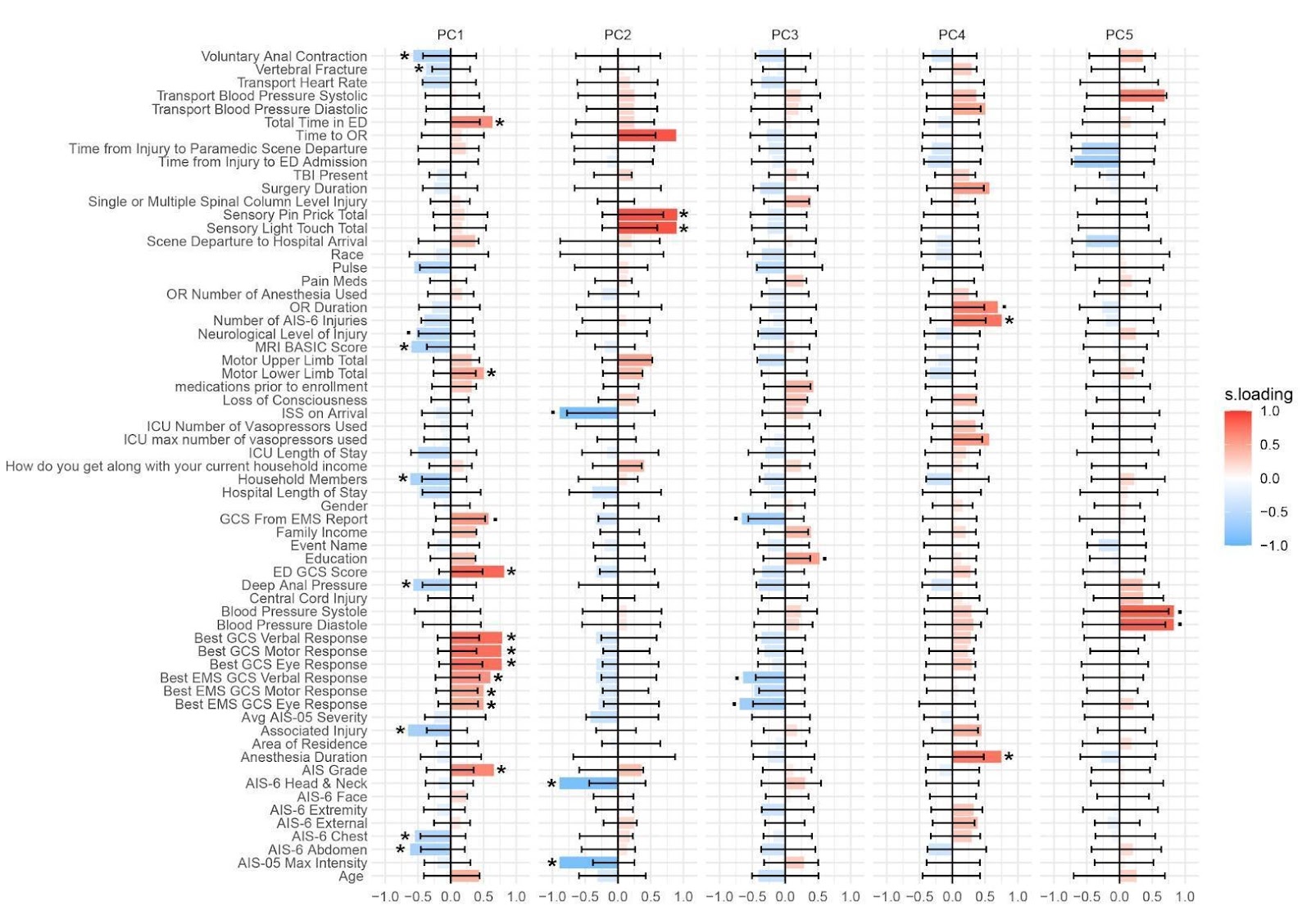


**Supplementary Figure 2.** Barmap plot of the variable loadings of the first 5 PCs after 1,000 permutations for each variable x PC. * p < 0.05, ^.^ p < 0.1


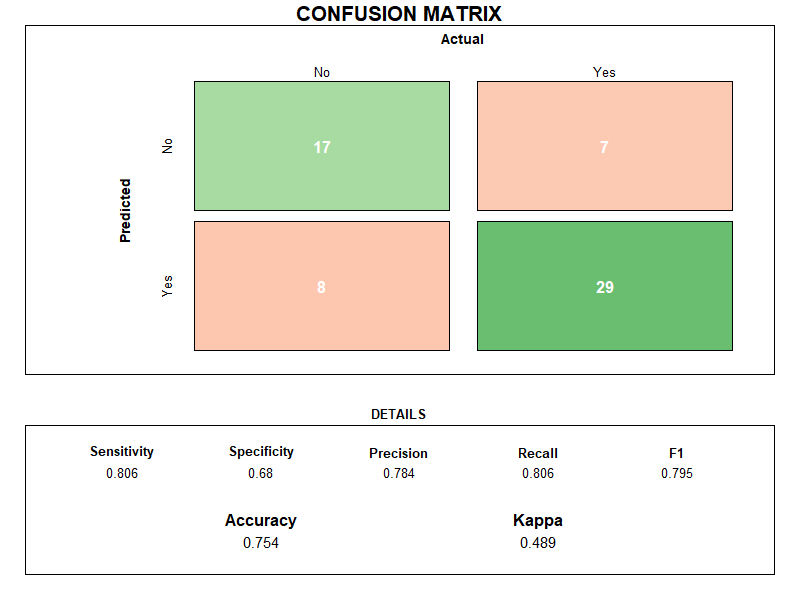


**Supplementary Figure 3.** Confusion matrix and model metrics.
